# Associations of Diet with Cardiometabolic and Inflammatory Profiles in Pregnant Women at Risk for Metabolic Complications

**DOI:** 10.3390/ijerph182111105

**Published:** 2021-10-22

**Authors:** Kataryna Jaworsky, Jeffrey L. Ebersole, Petar Planinic, Arpita Basu

**Affiliations:** 1 Department of Kinesiology and Nutrition Sciences, University of Nevada at Las Vegas, Las Vegas, NV 89154, USA; jawork1@unlv.nevada.edu; 2 Department of Obstetrics & Gynecology, Kirk Kerkorian School of Medicine, University of Nevada at Las Vegas, Las Vegas, NV 89154, USA; pplaninic@gmail.com; 3 School of Dental Medicine, University of Nevada at Las Vegas, Las Vegas, NV 89154, USA; jeffrey.ebersole@unlv.edu; 4 Valley View Hospital, Las Vegas, NV 89154, USA

**Keywords:** pregnancy, diet, obesity, total fats, fiber, inflammation

## Abstract

Dietary intakes play an important role in the development of metabolic complications during pregnancy. While reported observational studies reveal an inverse association of healthy diets with weight gain, gestational diabetes, and hypertensive complications during pregnancy, there is a paucity of studies conducted among women of specific ethnicities vulnerable to higher risks of pregnancy complications. This is a secondary cross sectional analysis using baseline data from a previously reported clinical trial. We aim to identify associations of maternal habitual dietary intakes with cardiometabolic risks and inflammatory profiles in primarily African American (AA) and Hispanic women in the first half of pregnancy. Fifty-two women met the study criteria and anthropometric, clinical, and dietary data were obtained at baseline. Linear regression analysis was used to determine associations after covariate adjustments. Among the maternal dietary nutrient intakes, total fats were positively associated with maternal body weight, BMI, and serum CRP (β ± SE: 0.25 ± 0.13, 0.28 ± 0.18, and 0.29 ± 0.14, respectively, all *p* < 0.05), and saturated fats were positively associated with glycated hemoglobin (0.32 ± 0.12). Dietary fiber intake showed a consistent inverse association with body weight (−0.26 ± 0.13), BMI (−0.19 ± 0.15), glycated hemoglobin (−0.22 ± 0.16), as well as serum CRP (−0.19 ± 0.14). Among the maternal food group intakes, dairy intake was inversely associated with systolic blood pressure (−0.18 ± 0.15) and serum IL-6 (−0.22 ± 0.17), and vegetable intake showed an inverse association with serum CRP (−0.17 ± 0.12) all in adjusted analyses (all *p* < 0.05). Thus, maternal diet modifications, especially decreasing fats and increasing fiber and dairy may help address obesity and inflammation leading to pregnancy complications in AA and Hispanic women.

## 1. Introduction

Metabolic complications during pregnancy, including gestational diabetes mellitus (GDM) and hypertensive disorders of pregnancy (HDP), can have serious consequences for both the mother and the child [1,2]. GDM is characterized as the onset of diabetes during pregnancy in a woman not previously diagnosed with diabetes [3]. HDP includes gestational hypertension, defined as hypertension after 20 weeks gestation, chronic hypertension, preeclampsia-eclampsia, and chronic hypertension with super-imposed preeclampsia [4]. GDM increases the mother’s risk for developing type 2 diabetes mellitus and the risk of additional pregnancy and birth complications, including preeclampsia [3]. GDM can also have negative effects on the child [5], such as macrosomia, vaginal delivery complications leading to shoulder dystocia, increased risk of diabetes later in life, and postnatal hypoglycemia [6]. HDP can result in cerebrovascular complications during pregnancy and delivery, including seizures or hemorrhage [7], and may cause maternal postpartum hypertension and premature cardiovascular disease (CVD) [8]. Fetal HDP complications consist of placental insufficiency, intrauterine growth restriction, oligohydramnios [9], placental abruption, and potential increased risk of CVD in later life [10]. HDPs are also a leading cause of maternal and fetal morbidity and mortality in the US [4]. GDM and HDP have similar prevalence at approximately 10% each [11,12], both of which have been increasing due to climbing obesity rates in women of childbearing age [11,13]. Due to the increasing prevalence of GDM and HDP, as well as the potentially severe consequences, dietary intake and modification during pregnancy have been of great interest in determining and implementing strategies for their reduction.

The risk factors that predispose a pregnant woman to metabolic complications are numerous, and early detection is of utmost importance to allow for management and prevention of future complications. These include modifiable risk factors, such as smoking, obesity, poor diet, and essential hypertension prior to conception, and non-modifiable risk factors, such as family history of GDM or diabetes, GDM or HDP in previous pregnancies, and advanced maternal age [14]. Ethnicity also plays an important role in determining metabolic risk- studies have shown that age-adjusted prevalence of GDM is higher in Hispanic and Asian American women compared to Caucasian women, suggesting a genetic determinant to GDM risk that varies between ethnicities [15]. Similarly, epidemiologic studies on HDP have showed increased overall prevalence in non-white ethnicities [16], with increased HDP-related mortality in African American pregnant women [17].

Obesity and concomitant poor dietary intake, some of the most important GDM and HDP risk factors, have been targeted by researchers in maternal fetal medicine. Obesity is a current public health crisis, and prevalence in the United States is increasing dramatically [11]. It has been shown that obese women (BMI ≥ 30 kg/m^2^) have higher rates of GDM compared to non-obese women, with a population-attributable fraction of GDM development due to being overweight/obese (BMI > 25 kg/m^2^) of 46.2% [18]. Furthermore, a meta-analysis of 20 studies showed a strong correlation between obesity and GDM diagnoses- obese women were 3.56 times and severely obese women were 8.56 times more likely to develop GDM compared to women with a normal pre-pregnancy BMI [19]. Effects of obesity were similar for development of HDP, as one observational study demonstrated that obesity resulted in women being 2.8 times more likely to develop gestational hypertension and 3.4 times more likely to develop preeclampsia [20]. Finally, both GDM and HDP risks are related to one another—in women with diagnosed GDM, pre-pregnancy obesity had an 88.8% attributable risk to the development of HDP [21].

The mechanism behind obesity conferring a heightened risk of GDM and HDP is of increasing importance to study, as maternal biomarkers such as C-reactive protein (CRP), adiponectin, plasminogen activating inhibitor-1 (PAI-1), tumor necrosis factor alpha (TNF-α), and interleukin-6 (IL-6) play an important role in this pathophysiology. Both GDM and HDP are characterized by hyperinsulinemia [22]; the cells become resistant to insulin action, so the pancreas secretes more in an attempt to achieve a normal response, which contributes to development of adiposity resulting from the anabolic action of insulin. This adiposity results in increased secretion of CRP, PAI-1, IL-6, and TNF-α, all pro-inflammatory cytokines, directly from fat deposits [23,24]. Decreased secretion of adiponectin, a hormone thought to be anti-inflammatory in its glucose regulation and fatty acid breakdown functions, is also seen in adiposity accretion [25]. The increased levels of pro-inflammatory markers, and decreased anti-inflammatory mediators, result in endothelial dysfunction, which can contribute to HDP and GDM development [24].

Diet plays a crucial role in the development of metabolic complications in pregnancy, both related to and independent of the mother’s pre-pregnancy BMI. One observational study showed that a low-carbohydrate, high animal protein, and high animal fat diet was positively associated with increased GDM risk, while a low-carbohydrate, high vegetable protein, and vegetable fat diet was not associated with increased GDM risk [26]. Low-carbohydrate diets, often thought to be beneficial in lowering diabetes risk, are often supplemented with increased dietary fats, especially animal fats [26]. Thus, supplementing diets with vegetable-based fats may be more beneficial in GDM risk reduction. Another study showed that women with GDM had significantly higher LDL cholesterol and triglycerides, along with lower HDL cholesterol, implying that high cholesterol and triglycerides, likely from poor dietary habits, increase the risk of GDM development [27]. Studies focusing on dietary influence of HDP have similarly shown conflicting results. One observational study documented lower risks of HDP in those following a Mediterranean diet (characterized by increased intake of vegetables, legumes, fish, and healthy fats) but reported no effect on HDP rates among women on diets high in meat, fats, sugar, or dairy [28]. Higher-than-median carbohydrate intake also showed an increased HDP prevalence compared to women who had lower-than-median carbohydrate intake [29]. Thus, it appears that both pre- and mid-pregnancy diets may modulate risks of developing GDM and HDP and should be further studied.

Although many studies have documented associations between obesity and the risk of metabolic complications of pregnancy including GDM and HDP, few have reported the associations of diet with specific cardiometabolic risk factors, especially maternal biomarkers of inflammation and adiposity in the first half of pregnancy in high-risk women. Thus, this cross-sectional analysis aims to examine the association between diet and specific biomarkers of inflammation (e.g., CRP, IL-6, adiponectin) in obese pregnant women with high risk of developing GDM and hypertensive complications.

## 2. Materials and Methods

### 2.1. Study Design

This is a cross sectional study using baseline data from women who qualified for a randomized controlled trial as reported previously [30]. All women (*n* = 52) provided informed consent and the study was approved by the Human Ethics Committee at the University of Nevada at Las Vegas (UNLV IRB#1155039). Women were recruited at the Department of Obstetrics and Gynecology at the UNLV School of Medicine based on criteria previously reported [30]. In brief, women were enrolled in the study if they had a BMI > 30 kg/m^2^ and had risks of GDM based on medical and pregnancy history, carrying a single fetus, and had gestational age < 20 weeks. All women had access to standard prenatal care and data were collected through face-to-face interviews with a nurse practitioner and a registered dietitian. Body weight, height, systolic and diastolic blood pressure were recorded during the baseline visit and involved averaging three measurements over 2 weeks of the study.

### 2.2. Dietary Data

Participants were asked to maintain a 3-day food record for at least 2 weeks in this cross-sectional study. These records included detailed intakes of all foods and beverages consumed by each participant for the entire day expressed in household measurements, such as cups and spoons to express the amounts consumed. Dietary analyses were conducted by the study RD or a trained dietetic assistant using ESHA’s Food Processor^®^ Nutrition Analysis software for energy, nutrients, and food group intakes for each participant. This is a comprehensive software and carries information on approximately 18,000 foods, beverages and other edible compounds to choose from. Sources of nutrient data in the ESHA database include the USDA Nutrient Database for Standard Reference and the USDA Database for the Continuing Survey of Food Intake by Individuals. Each food item was searched in the software database and the corresponding amount of intake per day was selected and added to each participant’s diet report for nutrient analyses. The average intakes based on these records over 2 weeks have been reported.

### 2.3. Clinical Variables and Serum Inflammatory Data

Freshly drawn blood samples were sent to Quest diagnostics (Las Vegas) for determination of serum HbA1c, triglycerides and HDL-cholesterol. Biomarkers of inflammation (CRP, IL-6, adiponectin, PAI-1 and TNF-α) were measured using a quantitative sandwich enzyme immunoassay technique (R&D Systems). The average intra-assay CVs for these analyses were in the range of 3–7%.

### 2.4. Statistical Analyses

Descriptive statistics were used to summarize data and baseline characteristics for continuous variables were expressed as means ± SDs and discrete variables as percentages. Our main objective for this cross-sectional analysis was to identify any significant association of maternal dietary intakes (nutrients and food groups) with body weight, blood pressure, and biomarkers of glycemic control, lipids, and inflammation. To address this, we employed a linear regression analysis in a multivariate model adjusted for relevant maternal covariates (age, race, prenatal vitamin use, and caloric intake). All *p* values were two-tailed, and significance was considered if *p* was *<* 0.05. Analyses were performed using SPSS version 26.0 (SPSS).

## 3. Results

Table 1 shows the baseline characteristics of the enrolled women (*n* = 52) who were consented and provided complete dataset. Overall, women met the criteria of obesity (BMI > 30 kg/m^2^), had above normal systolic blood pressure, serum triglycerides and CRP, but mean HbA1c in the normal range. Women were largely of self-reported Hispanic origin (~80%) and were at high risk of developing GDM based on history of GDM and family history of diabetes. Among the nutrient intakes, women reported 50% caloric intake as carbohydrates, 35% as dietary fats and the remaining 14% as proteins. Intake of fruits, vegetables, and dairy fell below the recommendations.

Table 2 presents the linear associations of dietary nutrient and food group intakes with cardiometabolic risks in women with obesity in first half of pregnancy in our study. Among the dietary nutrient intakes, we observed a significant positive association of total fats with maternal body weight and BMI, and a positive association of saturated fats with HbA1c (all *p* <0.05). As expected, dietary fiber showed an inverse association with body weight, BMI and HbA1c (*p* < 0.05). Among the food groups, dairy intake showed a protective association against elevated systolic blood pressure (*p* < 0.05). All analyses were adjusted for maternal age, race, prenatal vitamin use, and total caloric intake.

Table 3 presents linear associations of dietary nutrient and food group intakes with biomarkers of inflammation in women with obesity in first half of pregnancy in our study. Among the dietary nutrient intakes, we observed a significant positive association of total fats with maternal CRP (*p* < 0.05), but an inverse association of maternal fiber intake with CRP (*p* < 0.05). Among the food groups, dairy intake showed a protective association against elevated IL-6, and vegetable intake was inversely associated with CRP (all *p* < 0.05). All analyses were adjusted for maternal age, race, body mass index, prenatal vitamin use, and total caloric intake.

## 4. Discussion

In our study of obese pregnant women at increased risk for metabolic complications, we observed multiple significant associations of maternal habitual dietary intakes with cardiometabolic risks and inflammatory profiles in first half of pregnancy. Increased total fat intake was associated with an increased maternal body weight and BMI, while increased saturated fat intake was associated with an increased maternal HbA1c level. Increased fiber intake appeared to be protective, as it was inversely associated with maternal body weight, BMI, and HbA1c. Dairy also appeared to confer a protective effect on systolic blood pressure and IL-6 levels through an inverse association. Total fats, fiber, and vegetable intake were all significantly associated with maternal CRP levels, with increased total fat intake being positively associated, and increased fiber and vegetable intake being inversely associated with CRP. These findings demonstrate that dietary intake may have a role in determining maternal risk for metabolic complications, including GDM and HDP, during pregnancy.

High fat diets are of increasing public health concern in the United States, especially as both fat intake and obesity levels rise throughout all age groups [31]. Obesity is one of the most important modifiable risk factors for development of pregnancy metabolic complications, and therefore, diet modification is also of utmost importance. Our study showed that increasing total fat intake by 1 g is associated with a 0.25 kg increase in maternal body weight, a 0.28 kg/m^2^ increase in maternal BMI, and a 0.29 mg/L increase in serum CRP levels, all of which pose an increased risk for developing metabolic complications in pregnancy [32,33]. There appears to be mixed results in the literature regarding fat intake and resulting inflammatory profiles and risk for metabolic complications in pregnancy. In an observational study, Saldana et al. showed replacing carbohydrate intake with fat intake caused an increased risk for GDM and decreasing fat intake by 10% resulted in a decrease in probability of developing GDM [34]. These findings are similar to ours, although it is important to note that the women studied in this previous trial were not all obese (as classified by BMI). Sen et al. also demonstrated that a pro-inflammatory diet during pregnancy, characterized by high total fat intake, was positively associated with CRP levels as shown in our study, but showed no overall association with preeclampsia, suggesting that although there is a relation between fat intake and pro-inflammatory markers such as CRP, this association alone may not be strong enough to induce HDP complications of pregnancy [35]. Furthermore, our study showed that intake of saturated fats, defined as less healthy compared to unsaturated fats, specifically showed a positive association with HbA1c, with a 1-g increase in intake resulting in a 0.32% increase in HbA1c level. There are conflicting data in the literature regarding saturated fat intake and effects on pregnancy complications. Park et al.’s study highlighted that both normal weight and obese women diagnosed with GDM had higher saturated fat intakes compared to non-GDM women, suggesting that saturated fats may contribute to GDM development, showing similar results to our study [36]. Conversely, Qiao et al. showed no association between saturated fat intake with HbA1c levels and resulting risk of GDM development [37]. This could be explained by the differences in saturated fat intakes. In our study, women had an average daily saturated fat intake of 47 ± 18 g per day, which is much higher than the recommended daily average of 10% of caloric intake, while the median saturated fat intake for women in the study by Qiao et al. ranged from 7.7 to 18 g per day, which may explain the lack of association between these two studies [38]. All women in our study were obese and had higher than recommended levels of daily total fat and saturated fat intake. This increased fat intake can result in increased adiposity, which increases secretion of inflammatory markers such as CRP that can eventually lead to increased risk of GDM and HDP [39].

Dietary fiber has been shown in numerous studies to have positive effects on glycemic control in pregnant women. The results reported by Zhang et al. demonstrated that a 10 g per day increase in fiber intake was associated with a 26% reduction in GDM risk [40]. Our results illustrate a similar connection between increased fiber intake and reduction of classic risk factors for GDM and other metabolic complication in pregnancy; a 1 g increase in fiber intake was associated with a 0.26 kg decrease in body weight, a 0.19 kg/m^2^ decrease in BMI, a 0.22% decrease in HbA1c, and a 0.19 mg/L decrease in CRP. Dietary fiber has been shown to decrease gestational glucose intolerance and hypertensive risks during pregnancy, and can also assist with appropriate gestational weight gain, which can in turn mitigate effects of obesity during pregnancy and the resulting metabolic complications [41]. Fiber can be derived from many sources in the diet, including vegetables, which also showed an inverse association with serum CRP levels in our pregnant obese women; a 1 cup increase in vegetable intake resulted in a 0.17 mg/L decrease in serum CRP levels. Similarly, many studies have shown that vegetable intake can alter the risks of metabolic pregnancy complications, including the findings of Shin et al. and Zhang et al., who documented that decreased vegetable intake during pregnancy results in an increased risk of GDM, and Torjusen et al., who showed that women consuming a diet characterized by high organic vegetable intake had a decreased risk of preeclampsia [42,43,44]. In a study focused on the general population, Lahoz et al. demonstrated that diets high in vegetables (≥2 servings/day) are associated with decreased levels of CRP, which is pro-inflammatory and can lead to endothelial dysfunction, implying that vegetables aid in anti-inflammatory effects [45]. Increasing dietary fiber intake, and concomitant vegetable intake, should therefore be considered to reduce risks of developing metabolic complications during pregnancy.

Dairy intake also showed significant associations with unique maternal outcomes that were not associated with any other dietary intakes. Specifically, a 1 cup increase in dairy intake was associated with a 0.18 mmHg decrease in systolic blood pressure and a 0.22 pg/mL decrease in serum IL-6 levels in our study. IL-6 is a proinflammatory molecule secreted from adipose deposits and increased levels can lead to metabolic complications such as GDM and HDP. Therefore, decreasing the levels of IL-6 may be beneficial in improving these risks [33]. Moosavian et al., in a meta-analysis of randomized control trials in non-pregnant adults of varying health statuses, documented that high consumption of dairy products showed a significant reduction in inflammatory markers such as IL-6 [46]. However, in a study focused specifically on pregnant women and the risk of HDP, Schoenaker et al. showed that a diet focused on increased consumption of fruit and low-fat dairy products did not have an association with HDP incidence [28]. This may be because the analysis combined fruit and dairy as food groups which could mask results associated with dairy intake per se, as both fruits and low-fat dairy intake have been shown to individually influence HDP diagnoses [47]. Furthermore, a meta-analysis by Schoenaker, et al. found that higher calcium intake during pregnancy decreased risk of developing HDP. Thus, dairy, a large source of dietary calcium, could be used as potential calcium supplementation to provide protection against HDPs, as the results of our study suggest [48].

Interestingly, increased carbohydrate intake, often a target of nutritional modifications, in the maternal diet was not significantly associated with any of the cardiometabolic biomarkers measured in this study. While studies have shown relationships between increased carbohydrate intake and resulting risk of GDM and HDP, our study did not show such results [29,49]. This is likely because the daily carbohydrate intake of women enrolled in our study, which averaged at 272 ± 102 g/day and 50% of daily caloric intake, is not considered high carbohydrate intake, which most sources define as greater than approximately 60% of daily caloric intake [50]. Despite these results, it is still important to monitor carbohydrate intake in pregnant women to avoid excess gestational weight gain and obesity, which can lead to metabolic complications during pregnancy.

Our study has many strengths, one of the most important being the diversity of women enrolled in the clinical trial from which the data for this cross-sectional analysis were derived. Out of the 52 women enrolled, 81% were Hispanic and 19% were African American. Both ethnic groups are at higher risk for metabolic complications during pregnancy, including GDM and HDP, so having a study composed entirely of these high-risk women allows the results to be applicable to those who would be benefited the most [15,16]. In addition to ethnicity, the women enrolled in the study were also all at high-risk for metabolic complications based on their baseline maternal characteristics. All enrolled women were obese at baseline, with average BMI being 36 ± 4.2 kg/m^2^, which put them at high-risk for metabolic pregnancy complications. In total, 75% of individuals also had a history of GDM in past pregnancy, which also makes their current pregnancy a high-risk pregnancy. We also present associations of maternal dietary intakes with a combined panel of cardiometabolic, and inflammatory biomarkers not reported in other cross-sectional studies.

However, we also acknowledge certain limitations present within our study. Firstly, because this study is a cross-sectional analysis of baseline data from a previously reported clinical trial [30], we can only discuss correlations of dietary intakes with cardiometabolic and inflammatory profiles and not causality. Secondly, because our participants were all obese (BMI > 30 kg/m^2^) and before 20 weeks gestation when enrolled, there is a lack of generalizability of these results to the population of pregnant women who range from underweight to obese. Thirdly, none of these women were followed through post-pregnancy and into postpartum life or subsequent pregnancies, and thus associations cannot be speculated in these crucial phases of reproductive years. Finally, we did not analyze other exposures and outcomes, such as maternal fast food or convenience food intakes, or maternal or placental inflammatory cytokines and hormones, and these should be studied in the future. Despite these limitations, we believe our findings contribute to the investigation of dietary influences on cardiometabolic and inflammatory profiles in high-risk pregnant women and provide evidence for appropriate nutritional measures during pregnancy.

## 5. Conclusions

In conclusion, our study has shown that in obese women, primarily of Hispanic origin, maternal dietary intake modulates important cardiometabolic risks and inflammatory profiles during pregnancy. Increased total fat and saturated fat intake showed positive associations with classic risk factors for metabolic complications in pregnancy, especially maternal body weight, BMI, HbA1c, and CRP. Conversely, increased fiber, dairy, and vegetable intake appeared to be inversely related to these classic risk factors for metabolic complications. Increased fiber intake was associated with decreases in maternal body weight, BMI, HbA1c, and CRP, increased dairy intake resulted in decreased IL-6 and systolic blood pressure, and increased vegetable intake resulted in decreased maternal CRP levels, thus suggesting a protective effect of these in the diet. These dietary nutrients and food groups should be further studied in observational and clinical trials to determine causality between maternal dietary intake and metabolic complications in pregnancy.

## Figures and Tables

**Table 1:** Baseline maternal characteristics.

Variable	Value ^1^
Age, y	32 ± 4.2
Gestational age, weeks	15 ± 4.2
Body weight, kg	102 ± 4.4
BMI, kg/m^2^	36 ± 3.2
HbA1c, %	5.1 ± 4.7
Systolic blood pressure, mm Hg	131 ± 15
Diastolic blood pressure, mm Hg	78 ± 9
Serum triglycerides, mg/dL	204 ± 14
Serum HDL-cholesterol, mg/dL	58 ± 12
Serum CRP, mg/L	7.3 ± 5.2
Serum Adiponectin, µg/mL	10.4 ± 6.3
Serum IL-6, pg/mL	32 ± 11
Serum PAI-1, pg/mL	6121 ± 1032
Serum TNF-α, pg/mL	13 ± 6
Race	
African American, %	19
Hispanic, %	81
Prenatal vitamin users, %	42
History of GDM, %	75
Family history of diabetes, %	40
Nulliparous, %	32
Energy intake, kcal	2156 ± 834
Carbohydrates, g	272 ± 102
Total fats, g	85 ± 43
Saturated fats, g	47 ± 18
Proteins, g	75 ± 21
Fiber, g	12 ± 8
Vitamin C, mg	38 ± 21
Vitamin E, mg	12 ± 7
Calcium, mg	834 ± 81
Iron, mg	12 ± 5
Zinc, mg	8 ± 5
Dairy, cup	1.2 ± 0.4
Fruits, cup	0.5 ± 0.2
Vegetables, cup	1.4 ± 0.6

^1^ Values are means ± SD, *n* = 52. Race, prenatal vitamin users, history of GDM, family history of diabetes, and nulliparous are expressed as percentages. Abbreviations: BMI, body mass index; CRP, C-reactive protein; GDM, gestational diabetes mellitus; IL-6, interleukin-6; PAI-1, plasminogen activator inhibitor-1; TNF-α, tumor necrosis factor alpha.

**Table 2:** Linear associations of dietary nutrient and food group intakes with cardiometabolic risks in women with obesity in first half of pregnancy (*n* = 52).

	Maternal Outcomes						
Dietary Nutrients/Food Groups	Body Weight	BMI	HbA1c	SBP	DBP	TG	HDL-C
	β ± SE	β ± SE	β ± SE	β ± SE	β ± SE	β ± SE	β ± SE
Carbohydrates	0.14 ± 0.10	0.15 ± 0.08	0.22 ± 0.11	0.05 ± 0.01	0.02 ± 0.01	0.12 ± 0.07	0.15 ± 0.11
Total fats	**0.25 ± 0.13**	**0.28 ± 0.18**	0.18 ± 0.12	0.08 ± 0.01	0.05 ± 0.01	0.15 ± 0.08	0.11 ± 0.09
Saturated fats	0.16 ± 0.12	0.11 ± 0.12	**0.32 ± 0.12**	0.12 ± 0.05	0.08 ± 0.04	0.18 ± 0.05	0.10 ± 0.09
Proteins	0.11 ± 0.12	0.08 ± 0.10	0.16 ± 0.12	0.12 ± 0.05	0.05 ± 0.02	0.18 ± 0.12	0.09 ± 0.06
Fiber	**−0.26 ± 0.13**	**−0.19 ± 0.15**	**−0.22 ± 0.16**	0.11 ± 0.06	0.07 ± 0.04	0.14 ± 0.12	0.11 ± 0.08
Vitamin C	0.05 ± 0.02	0.08 ± 0.03	0.11 ± 0.12	0.07 ± 0.05	0.04 ± 0.02	0.13 ± 0.09	0.04 ± 0.06
Vitamin E	0.10 ± 0.08	0.05 ± 0.04	0.04 ± 0.01	0.11 ± 0.05	0.06 ± 0.03	0.11 ± 0.12	0.06 ± 0.06
Calcium	0.06 ± 0.05	0.08 ± 0.11	0.11 ± 0.12	0.09 ± 0.05	0.05 ± 0.02	0.12 ± 0.10	0.09 ± 0.11
Iron	0.01 ± 0.02	0.04 ± 0.03	0.10 ± 0.10	0.09 ± 0.06	0.03 ± 0.02	0.11 ± 0.12	0.07 ± 0.06
Zinc	0.05 ± 0.06	0.08 ± 0.10	0.13 ± 0.12	0.10 ± 0.07	0.05 ± 0.02	0.12 ± 0.11	0.09 ± 0.06
Dairy	−0.14 ± 0.13	−0.11 ± 0.14	0.15 ± 0.16	**−0.18 ± 0.15**	−0.11 ± 0.06	0.11 ± 0.12	0.09 ± 0.11
Fruits	0.08 ± 0.06	0.12 ± 0.10	0.09 ± 0.12	0.09 ± 0.07	0.05 ± 0.02	0.12 ± 0.09	0.05 ± 0.06
Vegetables	−0.13 ± 0.07	0.09 ± 0.10	0.10 ± 0.12	0.08 ± 0.07	0.04 ± 0.02	0.01 ± 0.02	0.04 ± 0.05

Adjusted for maternal age, race, prenatal vitamin use, and total caloric intake. Abbreviations: BMI, body mass index; SBP, systolic blood pressure; DBP, diastolic blood pressure; TG, serum triglycerides; HDL, serum HDL-cholesterol. Significant estimates with *p* < 0.05 are shown in bold font.

**Table 3:** Linear associations of dietary nutrient and food group intakes with biomarkers of inflammation in women with obesity in first half of pregnancy (*n* = 52).

	Maternal Outcomes				
Dietary Nutrients/Food Groups	CRP	Adiponectin	PAI-1	IL-6	TNF-α
	β ± SE	β ± SE	β ± SE	β ± SE	β ± SE
Carbohydrates	0.10 ± 0.08	0.11 ± 0.07	0.09 ± 0.11	0.04 ± 0.01	0.03 ± 0.01
Total fats	**0.29 ± 0.14**	0.11 ± 0.08	0.06 ± 0.04	0.08 ± 0.04	0.06 ± 0.03
Saturated fats	0.15 ± 0.13	0.08 ± 0.11	0.12 ± 0.12	0.07 ± 0.05	0.06 ± 0.04
Proteins	0.10 ± 0.09	0.11 ± 0.10	0.10 ± 0.12	0.13 ± 0.05	0.06 ± 0.02
Fiber	**−0.19 ± 0.14**	0.14 ± 0.12	0.12 ± 0.13	0.11 ± 0.07	0.07 ± 0.04
Vitamin C	0.04 ± 0.02	0.03 ± 0.03	0.10 ± 0.12	0.05 ± 0.04	0.04 ± 0.02
Vitamin E	0.12 ± 0.10	0.05 ± 0.04	0.02 ± 0.01	0.10 ± 0.06	0.05 ± 0.03
Calcium	0.04 ± 0.02	0.06 ± 0.07	0.11 ± 0.10	0.11 ± 0.09	0.06 ± 0.02
Iron	0.01 ± 0.01	0.05 ± 0.03	0.09 ± 0.10	0.05 ± 0.06	0.04 ± 0.02
Zinc	0.04 ± 0.03	0.07 ± 0.10	0.11 ± 0.12	0.09 ± 0.07	0.08 ± 0.10
Dairy	−0.10 ± 0.09	0.13 ± 0.12	0.08 ± 0.06	**−0.22 ± 0.17**	−0.12 ± 0.07
Fruits	0.12 ± 0.08	0.14 ± 0.10	0.06 ± 0.05	0.11 ± 0.07	0.04 ± 0.02
Vegetables	**−0.17 ± 0.12**	0.11 ± 0.10	0.13 ± 0.12	0.09 ± 0.07	0.03 ± 0.02

Adjusted for age, race, body mass index, prenatal vitamin use, and total caloric intake. Abbreviations: CRP, C-reactive protein; IL-6, interleukin-6; PAI-1, plasminogen activator inhibitor-1; TNF-α, tumor necrosis factor alpha. Significant estimates with *p* < 0.05 are shown in bold font.

## Data Availability

All generated data in this study are included in the article.
